# Elevated Systemic Inflammatory Response Index Is Associated With Increased Risk of Severe Acute Pancreatitis: A Systematic Review and Meta-Analysis

**DOI:** 10.7759/cureus.107928

**Published:** 2026-04-28

**Authors:** Sathish Narayanaswamy, Anastasia Postoev, Zartasha, Ashutosh Sharma, Himashi Gunaratne, Sonalben Chaudhary, Calvin R Wei, Neelum Ali

**Affiliations:** 1 Neurosciences, University Hospitals North Midlands, Stoke-on-Trent, GBR; 2 Internal Medicine, Caribbean Medical University, Willemstad, CUW; 3 Emergency Medicine, The University of Lahore Teaching Hospital, Lahore, PAK; 4 Internal Medicine, The Kathmandu Medical College, Kathmandu, NPL; 5 Medicine, University of Colombo, Colombo, LKA; 6 Internal Medicine, Zydus Sitapur Hospital, Sitapur, IND; 7 Research and Development, Shing Huei Group, Taipei, TWN; 8 Internal Medicine, University of Health Sciences Lahore, Lahore, PAK

**Keywords:** acute pancreatitis, disease severity, inflammatory biomarker, meta-analysis, systemic inflammation response index

## Abstract

Acute pancreatitis (AP) is a common gastrointestinal emergency with a significant proportion of patients progressing to severe acute pancreatitis (SAP), which carries substantial morbidity and mortality. A comprehensive search of the literature was performed in PubMed, Scopus, Web of Science, Embase, Cochrane Library, and Google Scholar, covering all records from database inception through February 2026. Eligible studies were those providing quantitative evidence on the association between the Systemic Inflammatory Response Index (SIRI) and the severity of acute pancreatitis (AP) in adult populations. Methodological rigor was evaluated using the Newcastle-Ottawa Scale, and statistical synthesis was conducted with a random-effects approach using RevMan software. A total of nine studies satisfied the inclusion criteria and were incorporated into the meta-analysis. The aggregated results indicated that higher SIRI values were significantly correlated with a greater likelihood of severe acute pancreatitis (odds ratio or OR: 1.99; 95% confidence interval or CI: 1.46-2.72). Furthermore, patients with severe AP exhibited markedly elevated mean SIRI levels compared with those with mild disease (mean difference or MD: 4.30; 95% CI: 0.41-8.19). Substantial heterogeneity was detected among the included studies (I² = 92% for the OR analysis and I² = 89% for the mean difference analysis). Publication bias was not assessed as the number of included studies was less than 10. The findings of this meta-analysis suggest that elevated SIRI at admission is associated with an increased risk of severe acute pancreatitis, with patients presenting with severe disease demonstrating higher SIRI values compared to those with mild pancreatitis. These results are hypothesis-generating and should be interpreted as preliminary evidence rather than definitive proof of clinical utility. Large-scale prospective studies are warranted to establish optimal cut-off values and validate their incremental utility over existing severity scores.

## Introduction and background

Acute pancreatitis (AP) is among the leading gastrointestinal conditions necessitating hospitalization, and its global incidence has risen consistently over the past decades [1]. Although most patients develop a mild and self-limited form of the disease, nearly 15-25% progress to moderately severe or severe acute pancreatitis (SAP). These advanced forms are linked to systemic complications, persistent organ failure, and mortality rates that may approach 30% [2,3].

Early and reliable identification of patients at risk for severe disease is essential to optimize management strategies and improve outcomes. Various clinical scoring systems and laboratory markers, such as the Ranson criteria, Acute Physiology and Chronic Health Evaluation II (APACHE II), the Bedside Index for Severity in Acute Pancreatitis (BISAP), systemic inflammatory response syndrome (SIRS) criteria, hematocrit, and C-reactive protein, have been developed to stratify disease severity [4,5]. Despite their widespread application, these tools often require multiple variables, may necessitate up to 48 hours for complete evaluation, and can demonstrate limited predictive performance during the early phase of presentation [6]. Consequently, there is increasing interest in identifying simple, accessible, and cost-efficient biomarkers capable of predicting severity at the time of admission.

Recently, inflammatory markers derived from routine complete blood count parameters have attracted growing attention as prognostic indicators in both acute and chronic diseases [7]. The Systemic Inflammation Response Index (SIRI), calculated as neutrophil count multiplied by monocyte count and divided by lymphocyte count, represents the interplay between pro-inflammatory and anti-inflammatory pathways [8]. First described in 2016 as a prognostic marker in patients with pancreatic cancer undergoing chemotherapy, SIRI has subsequently been investigated in multiple inflammatory and critical conditions, including coronary artery disease, ischemic stroke, and subarachnoid hemorrhage [9,10]. Because it is derived from standard hematological parameters, SIRI is particularly appealing for use in emergency departments and settings with limited resources. Unlike Neutrophil-to-Lymphocyte Ratio (NLR) and Platelet-to-Lymphocyte Ratio (PLR), which reflect only two immune cell interactions, SIRI incorporates monocyte count alongside neutrophil and lymphocyte counts, thereby capturing a broader dimension of innate immune activation that may offer superior prognostic performance in inflammatory conditions [7]

Recent studies indicate that SIRI may be a promising early predictor of AP severity. Several investigations have demonstrated significantly higher SIRI values in patients with SAP compared with those with mild AP, and some have reported favorable discriminative performance based on receiver operating characteristic curve analyses [11,12]. Nevertheless, variations in study design, sample size, patient characteristics, cut-off and outcome reporting limit the generalizability of individual findings, underscoring the importance of synthesizing the available evidence.

A systematic search of PubMed, Cochrane Library, and International Prospective Register of Systematic Reviews (PROSPERO) confirmed that no prior systematic review or meta-analysis has comprehensively examined the predictive utility of SIRI for determining the severity of AP. Accordingly, this study aims to systematically review and quantitatively synthesize existing evidence regarding the accuracy of SIRI in predicting SAP, thereby providing consolidated evidence to guide clinical decision-making and future research endeavors.

## Review

Methodology

Literature Search Strategy

A comprehensive systematic search was performed to identify studies evaluating the role of the SIRI in predicting the severity of AP. The following electronic databases were searched from their inception through February 2026: PubMed/MEDLINE, Scopus, Web of Science, Embase, and the Cochrane Library. In addition, Google Scholar was screened to identify grey literature and potentially relevant studies not indexed in major databases. The reference lists of all included articles and relevant reviews were also manually examined to ensure that no eligible studies were overlooked.

The search strategy incorporated both Medical Subject Headings (MeSH) and relevant free-text terms. Keywords included combinations of: “systemic inflammation response index,” “SIRI,” “acute pancreatitis,” “severe acute pancreatitis,” “pancreatitis severity,” “prognosis,” “prediction,” “outcome,” “biomarker,” and “inflammatory index.” Boolean operators (“AND” and “OR”) were used to appropriately combine search terms. A detailed search strategy is presented in the appendix. The search was limited to studies published in English; however, non-English articles with accessible English abstracts were screened for eligibility. The restriction to English-language publications was applied due to resource constraints in translation and may introduce language bias, as relevant studies published in other languages may have been missed. No restrictions were imposed on publication date to maximize coverage. The literature search was conducted independently by two reviewers.

Study Selection

Eligible studies were identified according to predefined inclusion and exclusion criteria established prior to the review process, following the Preferred Reporting Items for Systematic Reviews and Meta-Analyses (PRISMA) guidelines [13]. Studies were included if they met the criteria including adult patients (≥18 years) diagnosed with AP based on established diagnostic criteria, calculated SIRI using the standard formula (neutrophil count × monocyte count ÷ lymphocyte count), evaluated SIRI either as a continuous or categorical variable and reported outcomes as odds ratios (ORs), risk ratios (RRs), or mean differences (MD) between severity groups; and and employed an observational design, including retrospective cohort, prospective cohort, or cross-sectional studies.

Studies were excluded if they were case reports, case series, editorials, letters, conference abstracts, or review articles without original data. Additionally they were also excluded if they lacked extractable quantitative data linking SIRI to AP severity. Studies focused exclusively on pediatric populations or examined AP secondary to procedural causes such as endoscopic retrograde cholangiopancreatography (ERCP) were not included in this meta-analysis

There were two stages to the study screening process. First, all retrieved records' titles and abstracts were examined separately to weed out publications that were blatantly unnecessary. Second, the complete texts of studies that might be eligible were thoroughly evaluated in relation to the predetermined standards. Any disagreements were settled by conversation, and where needed, a third reviewer was consulted. A PRISMA flow diagram was used to describe and summarize the study selection procedure.

Data Extraction

A standardized data extraction form was designed and pilot-tested before formal data collection. Two reviewers independently extracted data, and discrepancies were resolved by consensus or third-party adjudication. Extracted information included study characteristics (first author, publication year, country, and study design), participant characteristics (sample size, age, sex distribution, and etiology of AP), criteria used to classify AP severity, such as the revised Atlanta Classification or other validated systems, details regarding SIRI calculation and timing of measurement in relation to hospital admission and relevant outcome data.

When multiple subgroups or time points were reported, data corresponding to admission SIRI values and overall AP severity were preferentially extracted. If necessary data were not directly provided, values were derived from tables, figures, or supplementary materials whenever possible.

Quality Assessment

The Newcastle-Ottawa Scale (NOS) was used to assess the methodological quality of the included studies. With a maximum score of nine stars, this method evaluates research in three areas: participant selection, study group comparability, and outcome assessment. A study was rated as high quality if it received at least seven stars, moderately good if it received five or six stars, and low quality if it received less than five stars [14].

Statistical Analysis

Data synthesis was carried out using Review Manager (RevMan 5.4.1, The Cochrane Collaboration, London, England, UK) software. The association between SIRI and SAP was estimated by calculating combined ORs with corresponding 95% confidence intervals (CIs). When comparing average SIRI values between different severity categories, pooled MDs and their 95% CIs were determined.

Given the expected variability in study design and patient characteristics, a random-effects model was employed. Statistical significance was defined as a two-sided p-value below 0.05. Between-study heterogeneity was assessed using the I² statistic. To evaluate the robustness of the overall results, a leave-one-out sensitivity analysis was conducted, whereby each study was removed sequentially to determine its impact on the pooled effect estimates.

Results

**Figure 1:**
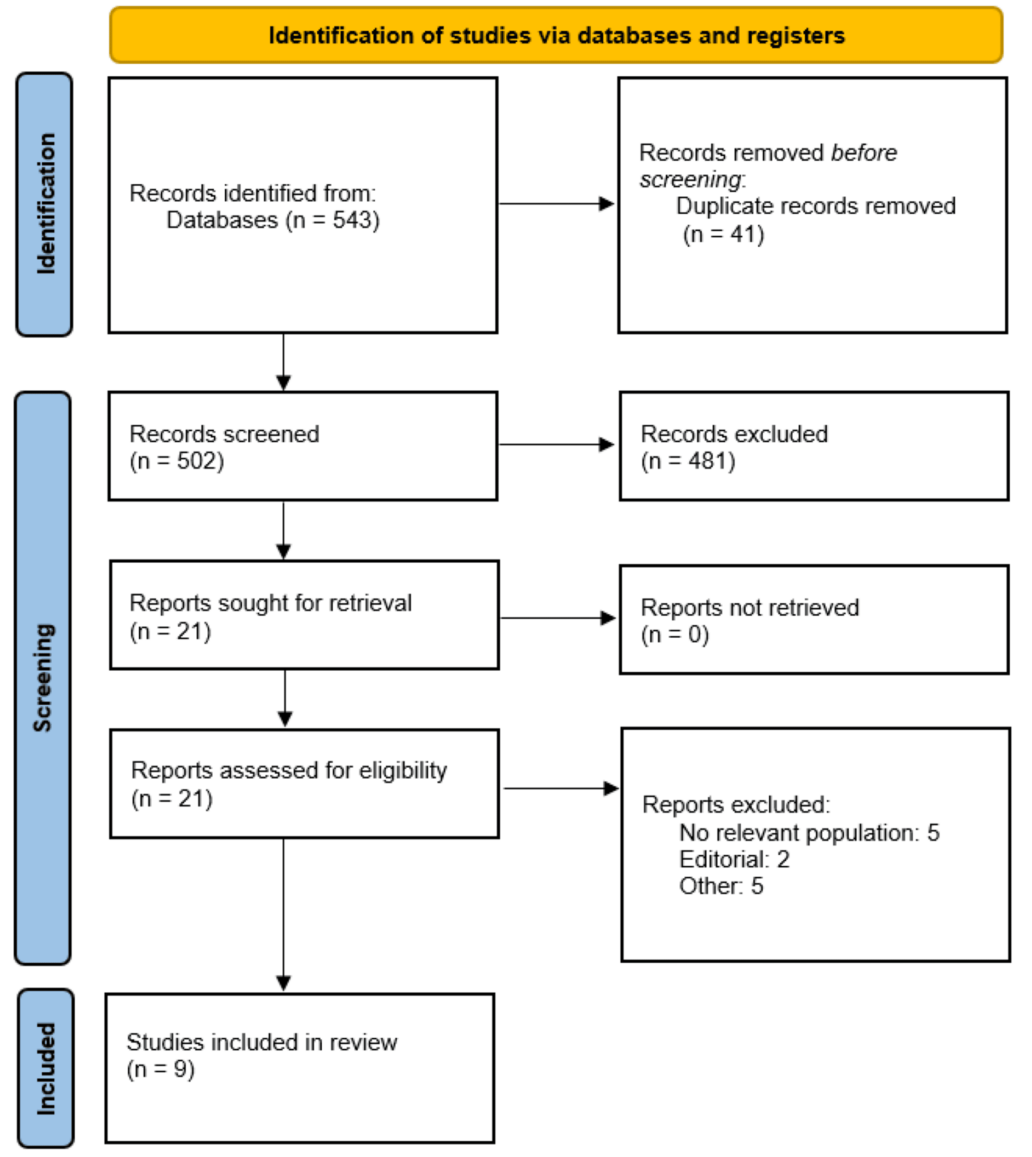
PRISMA flowchart PRISMA: Preferred Reporting Items for Systematic Reviews and Meta-Analyses.

There were 543 records found in the first database search. 502 papers were left for title and abstract screening after duplicate entries were eliminated. Twenty-one of them were deemed potentially eligible and were subjected to full-text review. Nine articles were finally included in the meta-analysis after a thorough evaluation based on the predetermined inclusion and exclusion criteria. Figure 1 (PRISMA flow diagram) depicts the study selection procedure.

Table 1 summarizes the characteristics of the selected studies.

**Table 1:** Included studies characteristics NR: Not reported; AP: Acute Pancreatitis; SIRI: Systemic Inflammatory Response Index.

Author	Year	Design	Region	Sample size	Number of subjects with severe AP	Cut-off used	Mean age	Males (n)	SIRI assessed
Araiza-Rodríguez et al. [15]	2025	Retrospective	Mexico	100	41	4.56	NR	75	At admission
Canlıkarakaya et al. [16]	2025	Retrospective	Turkey	213	37	NR	65.9	79	At admission
Dao et al. [17]	2025	Prospective	Vietnam	207	44	7.82	45.6	131	At admission
Gu et al. [18]	2025	Retrospective	China	343	76	NR	47	239	At admission
Huang et al. [19]	2025	Retrospective	China	592	31	4.27	43	411	Within 24 hours of admission
Li et al. [20]	2024	Prospective	China	253	60	NR	45.98	162	At admission
Ma et al. [21]	2026	Retrospective	China	1981	923	NR	NR	NR	At admission
Wu et al. [22]	2025	Retrospective	China	1514	171	4.83	56	799	At admission
Yildiz et al. [23]	2023	Retrospective	Turkey	201	36	9.5	NR	NR	At admission

The quality evaluation of the included studies is shown in Table 2.

**Table 2:** Quality assessment of included studies

Author	Selection	Comparison	Assessment	Overall
Araiza-Rodríguez et al. [15]	3	2	2	Good
Canlıkarakaya et al. [16]	2	1	2	Fair
Dao et al. [17]	3	2	2	Good
Gu et al. [18]	3	2	2	Good
Huang et al. [19]	4	2	2	Good
Li et al. [20]	3	1	2	Good
Ma et al. [21]	4	2	2	Good
Wu et al. [22]	4	2	1	Good
Yildiz et al. [23]	3	2	2	Good

Effect of High SIRI on the Severity of AP

**Figure 2:**
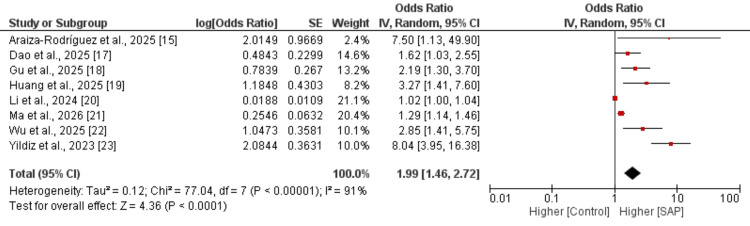
Effect of SIRI on severity of SAP SAP: Severe Acute Pancreatitis; SIRI: Systemic Inflammatory Response Index. [15,17-23].

Eight studies were included in the pooled analysis evaluating the association between elevated SIRI and the risk of SAP (Figure 2).

The pooled results demonstrated that higher SIRI levels were significantly associated with an increased likelihood of SAP (OR: 1.99, 95% CI: 1.46-2.72). Substantial heterogeneity was observed across the included studies.

Sensitivity analysis was conducted using a leave-one-out approach, with findings presented in Table 3.

**Table 3:** Sensitivity analysis

Author	OR (95% CI)	I-Square
Araiza-Rodríguez et al. [15]	1.92 (1.41 to 2.61)	92%
Dao et al. [17]	2.07 (1.48 to 2.90)	92%
Gu et al. [18]	1.95 (1.41 to 2.69)	91%
Huang et al. [19]	1.89 (1.38 to 2.58)	91%
Li et al. [20]	2.63 (1.59 to 4.35)	84%
Ma et al. [21]	2.61 (1.42 to 4.80)	91%
Wu et al. [22]	1.89 (1.38 to 2.60)	91%
Yildiz et al. [23]	1.60 (1.23 to 2.09)	87%

The results remained consistent after sequential exclusion of individual studies, indicating that no single study had a disproportionate impact on the overall effect estimate. Despite the presence of considerable heterogeneity, the stability of the pooled results supports the robustness of the observed association.

Comparison of Mean SIRI Levels Between Severity Groups

**Figure 3:**
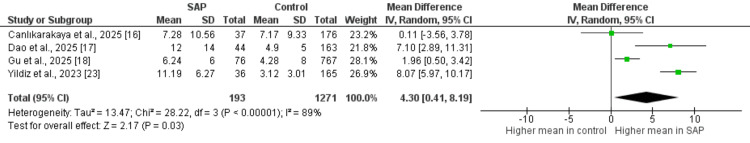
Mean SIRI score between two groups SIRI: Systemic Inflammatory Response Index. [16-18,23]

An additional analysis compared mean SIRI values between patients with SAP and those with non-severe disease (Figure 3).

Using a random-effects model, the pooled mean difference indicated that SIRI levels were significantly higher in patients with SAP (MD: 4.30, 95% CI: 0.41-8.19). However, high heterogeneity was also noted in this analysis (I² = 89%).

Discussion

This meta-analysis systematically assessed the relationship between the SIRI and the severity of AP. The pooled results suggest that higher SIRI levels are associated with a greater likelihood of SAP, though the substantial heterogeneity observed across included studies warrants cautious interpretation of the precise magnitude of this association.

SIRI was initially introduced by Qi et al. in 2016 as a prognostic indicator in oncology [8]. It integrates three key immune cell components - neutrophils (NEUs), monocytes (MONOs), and lymphocytes (LYMs) - into a single composite index. By simultaneously reflecting innate inflammatory activation (neutrophils and monocytes) and adaptive immune regulation (lymphocytes), SIRI offers a broader representation of systemic immune balance compared with single-parameter markers. Previous research has demonstrated its prognostic relevance in malignancies [24,25], and more recent investigations have extended its application to cardiovascular diseases, chronic obstructive pulmonary disease, coronavirus infection, and other inflammatory conditions [26,27].

The observed association between elevated SIRI and SAP is biologically plausible and consistent with the known immunopathology of the disease. AP is characterized by an exaggerated inflammatory response triggered by premature activation of pancreatic enzymes, leading to local tissue injury and systemic inflammatory activation [2]. In severe cases, this process progresses to SIRS, multi-organ dysfunction syndrome (MODS), and pancreatic necrosis [28]. Neutrophils, a dominant component of the SIRI formula, are among the earliest responders to pancreatic injury and play a central role in amplifying inflammation [29]. Their infiltration into pancreatic tissue results in the release of reactive oxygen species, proteolytic enzymes, and neutrophil extracellular traps (NETs), which contribute to acinar cell damage and distant organ injury [30,31]. Experimental models further support this mechanism, demonstrating that inhibition of NET formation attenuates pancreatitis severity and improves survival [31]. Thus, an elevated neutrophil count within the SIRI construct likely reflects heightened innate immune activation associated with more severe disease.

The findings of this meta-analysis should be interpreted in the context of existing evidence on inflammatory indices in AP. Prior pooled analyses evaluating the NLR have reported pooled ORs in the range of 2.5-3.5 for predicting SAP [32,33], suggesting that the magnitude of association observed for SIRI (OR: 1.99) is broadly comparable, though potentially more modest. Unlike NLR and PLR, however, SIRI incorporates the monocyte count, adding an additional dimension of innate immune activity. Monocytes and macrophages are critically involved in the early inflammatory phase of AP, influencing whether the inflammatory response remains localized or progresses systemically [34]. The added contribution of monocyte-driven inflammation may partly explain the prognostic performance of SIRI observed in various non-pancreatic inflammatory conditions [10,35], and the present findings suggest that this advantage may extend to AP. Nevertheless, without head-to-head diagnostic accuracy comparisons within the same patient cohorts, the incremental predictive value of SIRI over NLR and PLR cannot be definitively established from the current evidence.

From a clinical perspective, SIRI offers several practical advantages over established severity scoring systems such as the Revised Atlanta Classification, BISAP, Ranson criteria, and APACHE II, which may require multiple clinical parameters, serial assessments, or imaging studies not immediately available at admission [2,28]. SIRI is derived solely from a routine complete blood count, making it inexpensive, rapidly obtainable, and universally accessible. However, the clinical applicability of SIRI should currently be considered preliminary and hypothesis-generating rather than practice-ready. A critical barrier to immediate implementation is the considerable variability in reported SIRI cut-off values across included studies, ranging from 4.27 to 9.5, which prevents determination of a standardized clinical decision threshold. Until prospective studies establish and validate an optimal cut-off, SIRI should be regarded as a potentially useful supplementary indicator rather than a standalone triage tool.

The substantial heterogeneity observed in the pooled analyses (I² approaching 90%) deserves careful consideration and should not be treated as a statistical footnote. This level of variability substantially reduces confidence in the precision of the pooled estimates and reflects meaningful differences across included studies, including variations in patient demographics, geographic distribution, etiological patterns of AP, timing of SIRI measurement, and thresholds used to define elevated SIRI. Differences in institutional management protocols and severity classification criteria likely contributed further to this variability. Although the use of a random-effects model appropriately accounted for between-study differences, the high heterogeneity means that the pooled OR of 1.99 and MD of 4.30 should be interpreted as approximate estimates of a likely variable true effect rather than precise, universally applicable values.

Several potential sources of bias warrant acknowledgment. The predominantly retrospective observational design of included studies introduces the risk of selection bias and residual confounding from unmeasured variables including comorbidities, hematological conditions, corticosteroid use, and concurrent infections, all of which may independently influence SIRI values. The absence of individual patient-level data precluded adjustment for such confounders. Furthermore, the predominance of studies conducted in Asian populations, particularly from China, raises questions regarding the external validity of findings in Western settings, where differences in patient demographics, disease etiology, and management protocols may modify the relationship between SIRI and AP severity. Publication bias could not be formally assessed given the small number of included studies, representing an additional source of uncertainty.

Despite these limitations, this meta-analysis provides the most comprehensive synthesis to date of evidence linking SIRI with AP severity. The biological plausibility of the association, combined with the consistent direction of pooled findings across sensitivity analyses and the practical accessibility of SIRI, supports its potential as a supplementary prognostic marker worthy of further investigation. Future large-scale prospective multicenter studies with standardized SIRI measurement protocols, predefined cut-off values, and direct head-to-head comparisons against established severity scores and biomarkers such as CRP and procalcitonin are needed to clarify the incremental predictive value of SIRI and determine its optimal role in early risk stratification for AP.

## Conclusions

The findings of this meta-analysis suggest that elevated SIRI at admission is associated with an increased risk of SAP, with patients presenting with severe disease demonstrating higher SIRI values compared to those with mild pancreatitis. These results are hypothesis-generating and should be interpreted as preliminary evidence rather than definitive proof of clinical utility. Given the very high heterogeneity observed across included studies, the exclusively observational study designs, variability in SIRI cut-off values, and inconsistency in measurement timing, the pooled estimates warrant cautious interpretation. Furthermore, the incremental predictive value of SIRI over established severity scoring systems such as BISAP, APACHE II, and NLR has not been quantitatively assessed in this analysis, and direct comparative evidence is currently lacking.

Notwithstanding these constraints, SIRI is derived entirely from a routine complete blood count, making it a simple, cost-effective, and universally accessible tool that may complement - rather than replace - existing assessment frameworks, particularly in resource-limited settings where complex scoring systems are not readily applicable. Large-scale prospective multicenter studies employing standardized measurement protocols, uniform severity definitions, and head-to-head comparisons with established biomarkers are needed before any clinical recommendations regarding its routine incorporation can be made.

## Appendices

**Table 4:** Search strategy

Database	Search String
PubMed/MEDLINE	("systemic inflammation response index"[MeSH Terms] OR "systemic inflammation response index"[Title/Abstract] OR "SIRI"[Title/Abstract]) AND ("acute pancreatitis"[MeSH Terms] OR "severe acute pancreatitis"[Title/Abstract] OR "pancreatitis severity"[Title/Abstract]) AND ("prognosis"[MeSH Terms] OR "prediction"[Title/Abstract] OR "outcome"[Title/Abstract] OR "biomarker"[Title/Abstract] OR "inflammatory index"[Title/Abstract])
Scopus	TITLE-ABS-KEY("systemic inflammation response index" OR "SIRI") AND TITLE-ABS-KEY("acute pancreatitis" OR "severe acute pancreatitis" OR "pancreatitis severity") AND TITLE-ABS-KEY("prognosis" OR "prediction" OR "outcome" OR "biomarker" OR "inflammatory index")
Embase	('systemic inflammation response index'/exp OR 'systemic inflammation response index':ab,ti OR 'SIRI':ab,ti) AND ('acute pancreatitis'/exp OR 'severe acute pancreatitis':ab,ti OR 'pancreatitis severity':ab,ti) AND ('prognosis'/exp OR 'prediction':ab,ti OR 'outcome':ab,ti OR 'biomarker':ab,ti OR 'inflammatory index':ab,ti)
Web of Science	TS=("systemic inflammation response index" OR "SIRI") AND TS=("acute pancreatitis" OR "severe acute pancreatitis" OR "pancreatitis severity") AND TS=("prognosis" OR "prediction" OR "outcome" OR "biomarker" OR "inflammatory index")
